# Editorial: Debates in experimental pharmacology and drug discovery 2023: innovative approaches to chronic kidney disease drug discovery, identification of targets and safety assessment

**DOI:** 10.3389/fphar.2025.1588367

**Published:** 2025-03-25

**Authors:** Md Abdul Hye Khan, Omar Z. Ameer, Samaneh Goorani, Ibrahim M. Salman

**Affiliations:** ^1^ Department of Pharmaceutical Sciences, College of Pharmacy, University of Arkansas for Medical Sciences, Little Rock, AR, United States; ^2^ Department of Pharmaceutical Sciences, College of Pharmacy, Alfaisal University, Riyadh, Saudi Arabia; ^3^ Department of Pediatrics, School of Medicine, Case Western Reserve University, Cleveland, OH, United States

**Keywords:** chronic kidney disease, experimental pharmacology, drug discovery, novel target, translational mechanisms

Chronic kidney disease (CKD) remains a major global health challenge, with an increasing prevalence driven by associated comorbidities such as diabetes and hypertension. Despite advances in medical research, effective therapeutic strategies remain elusive, necessitating the exploration of innovative approaches. CKD is a progressive condition characterized by structural and functional kidney deterioration, ultimately leading to end-stage renal disease (ESRD). Current treatment modalities primarily focus on symptom management and slowing disease progression rather than reversing kidney damage. Thus, identifying novel drug targets and refining safety assessment strategies are crucial for advancing CKD treatment.

This Research Topic delves into diverse and promising strategies for CKD drug discovery, highlighting recent breakthroughs in pharmacological interventions, target identification, and safety evaluation. The studies featured in this Research Topic span a range of approaches, from traditional herbal medicine to advanced molecular and regenerative therapies, all of which providing valuable insights into CKD pathophysiology and potential treatment paradigms.

Natural compounds have always gained attention in nephroprotection. In a meta-analysis including 14 randomized controlled trials, with a total of 800 patients, Li et al. examined the synergistic effects of traditional Chinese medicine (TCM) and Western pharmacology in lupus nephritis (LN), a severe complication of systemic lupus erythematosus (SLE). LN is characterized by immune-mediated kidney inflammation, often leading to renal impairment. The paper revealed that combining Astragalus-containing Chinese herbal medicine (CHM) with standard treatments significantly improved renal outcomes while reducing adverse effects. This aligns with the growing body of literature supporting integrative medicine for complex diseases like LN. The findings also highlight the potential of TCM as an adjunctive therapy, optimizing treatment efficacy and safety in CKD patients.

Continuing with the natural products theme, an experimental study by Zeweil et al. elegantly investigated the renoprotective effects of *Annona muricata* (Graviola) against nephrotoxicity induced by 7,12-dimethylbenz(a)anthracene (DMBA), a harmful environmental pollutant and polycyclic aromatic hydrocarbon derivative known for its cytotoxic, carcinogenic, and mutagenic effects. Nephrotoxicity remains a major concern in CKD due to toxins, drugs, and environmental pollutants. This study demonstrated that Graviola significantly improved renal function by reducing oxidative stress and inflammatory markers, reinforcing the potential integration of natural products into CKD therapeutic strategies.

Xu et al. made a pivotal contribution by analyzing 2,409 CKD-related publications over 16 years, highlighting the critical link between mental health and CKD. Depression is prevalent among CKD patients and significantly impacts disease progression, quality of life, and treatment adherence. This bibliometric study identified key research hotspots, demonstrating that depression significantly affects CKD progression, quality of life, and mortality. The analysis stressed the need for comprehensive CKD management strategies that integrate psychological and physiological interventions, reinforcing the importance of a holistic treatment paradigm.

The study by Lu et al. focused on diabetic kidney disease (DKD), a leading cause of end-stage renal failure. DKD results from chronic hyperglycemia-induced renal damage, characterized by inflammation, oxidative stress, and fibrosis. Using transcriptome data from the Gene Expression Omnibus Database, the research team identified DNA damage-inducing transcription factor 4 (DDIT4) in human renal tissue as a key player within the vitamin D receptor (VDR)-mechanistic target of rapamycin (mTOR) pathway. Modulating DDIT4 expression in both DKD patients and DKD animal models appeared to alleviate oxidative stress and enhance autophagic activity, presenting a promising therapeutic target for DKD. This study not only contributes to our understanding of DKD pathophysiology but also offers a potential molecular target for future pharmacological interventions.

In the realm of regenerative experimental medicine, Khamis et al. evaluated the therapeutic potential of bone marrow-derived mesenchymal stem cells (BM-MSCs) in diabetic nephropathy (DN). DN is a severe complication of diabetes that leads to progressive kidney dysfunction. The findings revealed that BM-MSC therapy significantly reduced renal apoptosis, inflammation, and fibrosis, which suggested that stem cell therapy may be a viable intervention for DN. This study highlights the promise of regenerative medicine in CKD treatment, particularly for conditions involving irreversible renal damage.

Another unique approach to renal fibrosis treatment was explored by Mu et al. Their study introduced Nephropathy 1st, a traditional Chinese herbal formulation, and its impact on renal fibrosis. Renal fibrosis is a critical factor in CKD progression, characterized by excessive extracellular matrix deposition and scarring. By activating the peroxisome proliferator-activated receptor gamma (PPARγ) signaling pathway, the treatment seems to effectively mitigate fibrosis in a rat model of kidney injury. This research supports the potential of herbal medicine in modulating key molecular pathways involved in CKD progression, thus offering a foundation for future translational studies.

The final study in this Research Topic was conducted by Xu et al., employing a multi-omics approach to investigate the therapeutic potential of *Salvia miltiorrhiza* (Danshen), a potential adjunctive remedy in the treatment of diabetic microangiopathy, in DKD. Multi-omics techniques, including metabolomics, transcriptomics, and proteomics, provide comprehensive insights into disease mechanisms. The study identified critical pathways, including TGF-β/Smad and PI3K/Akt/FoxO, that are modulated by Danshen’s bioactive compounds. The findings of this study demonstrated the power of multi-omics in uncovering complex disease mechanisms and identifying effective therapeutic strategies.

Collectively, these studies illustrate the breadth of innovative approaches currently being explored in CKD drug discovery. The integration of TCM, stem cell therapy, natural compounds, and molecular targeting is reshaping nephrology research. Future investigations should focus on translating these findings into clinical applications, optimizing drug efficacy while minimizing adverse effects. Additionally, multi-omics and precision medicine approaches hold promise for redefining CKD treatment by enabling personalized therapeutic strategies.

As the field advances, a key challenge remains the validation and regulatory approval of novel CKD therapies. Ensuring the safety and efficacy of new interventions requires rigorous preclinical and clinical assessments, alongside robust translational frameworks. Collaboration between pharmacologists, nephrologists, and regulatory agencies will be essential in bringing these promising therapies from bench to bedside.

